# Decoupled quality and readability in skin cancer education from large language models

**DOI:** 10.3389/fpubh.2026.1777577

**Published:** 2026-02-20

**Authors:** Yanping Zhang, Lei Wang, Weiqiang Zhang, Weifeng Lan

**Affiliations:** 1 Department of Plastic Surgery, Longyan First Affiliated Hospital of Fujian Medical University, Longyan, China; 2 Department of Science and Education, Longyan First Hospital, Longyan, China

**Keywords:** digital public health communication, health information quality (C-PEMAT, GQS), large language models (LLMs), readability assessment, skin cancer education

## Abstract

**Introduction:**

Large language models (LLMs) are increasingly used by the public to obtain health information, yet the relationship between content quality and readability in LLM-generated patient education remains unclear.

**Methods:**

We benchmarked five LLMs (Doubao, DeepSeek, Wenxin Yiyan, Tongyi Qianwen, and GPT-5) using an identical set of 20 Mandarin Chinese skin-cancer FAQs (100 total outputs). Quality was assessed using c-PEMAT-P and the Global Quality Scale (GQS), and readability was assessed using seven indices (ARI, FRES, GFOG, FKGL, CL, SMOG, and LW). Group differences and correlations were evaluated with appropriate statistical tests.

**Results:**

Models showed comparable understandability/actionability (c-PEMAT-P), while overall quality (GQS) differed, with GPT-5 scoring highest. Readability varied substantially by both model and content category, and no single model performed best across all readability metrics. Correlation analyses indicated that quality and readability were largely decoupled.

**Discussion:**

High-quality outputs do not necessarily have high readability. Optimizing AI-generated skin-cancer education requires multi-faceted strategies that jointly consider model choice and content topic.

## Introduction

Public health education, essential for disease prevention and management, relies on disseminating information that is accurate, actionable, and accessible across diverse audiences (1). Skin cancer, a globally prevalent malignancy, illustrates this requirement, given that public knowledge directly influences early detection and subsequent outcomes (2, 3). Health communicators have long faced challenges balancing the dual imperatives of information quality—encompassing clarity, utility, and reliability—and textual readability, which governs cognitive accessibility (4). Successfully developing materials that meet both these stringent criteria demands significant expertise and iterative refinement.

### Target audience, language context, and model selection

This study focuses on Chinese-language (Mandarin) skin cancer educational materials intended for lay readers and patients, reflecting a common real-world scenario in which the public uses consumer-facing LLMs to obtain health information. We selected Doubao, DeepSeek, Wenxin Yiyan, and Tongyi Qianwen as representative publicly accessible LLM products widely available to Chinese users, and included GPT-5 as a widely used international reference model to enable cross-ecosystem comparison under identical prompts. Given the Chinese-language outputs, we employed the Chinese version of the Patient Education Materials Assessment Tool for Printable Materials (c-PEMAT-P) to support linguistically appropriate quality evaluation.

Generative Large Language Models (LLMs) offer unprecedented automation for creating health education content at scale (5). While their text-generation capacity is evident, their responsible deployment in healthcare necessitates rigorous, multidimensional validation (5). A pivotal question is whether LLMs consistently achieve the requisite integration of substantive quality and public readability (6). Existing research typically examines these metrics in isolation, often assuming correlation or treating them as a unitary construct (7, 8). This approach leaves a critical gap: the lack of systematic investigation into the explicit relationship between the quality and readability of LLM-generated health information. Without this understanding, the promise of LLMs in public health communication remains uncertain, creating a potential hazard since deficiencies in either dimension undermine essential educational objectives.

The pressing demand for reliable, accessible skin cancer information in dermatology drives interest in LLMs as a scalable source for generating consistent educational content (6–8). Initial LLM benchmarking utilizes standardized instruments—such as the c-PEMAT-P (for understandability/actionability) and the GQS (Global Quality Scale, for overall quality)—alongside readability formulas (e.g., Flesch–Kincaid, SMOG) (1, 9). However, the prevailing trend is to report these metrics separately, yielding a fragmented view of performance (10). The fundamental and largely overlooked problem concerns the inherent link between these evaluative axes: Does superior quality inherently correlate with improved readability, or are they distinct characteristics influenced independently by model choice and content subdomain? Resolving this empirical gap is critical for developing optimized deployment protocols. If coupled, quality benchmarking suffices; if decoupled (the central hypothesis of this study), the strategy requires nuance, as high factual quality may yield overly complex text, and vice versa. Thus, a focused analysis is essential to disentangle the effects of model architecture, content topic, assessed quality, and measured readability within AI-generated skin cancer education. While large language models (LLMs) show promise in generating high-quality content for public health, it is essential to address the risks of hallucinations and biases inherent in these models. Such issues could result in the spread of incorrect or biased information, which may compromise the reliability of the generated content.

### Quality versus readability and clinical expectations

In this study, content quality refers to the clarity, usefulness/actionability, and perceived reliability of patient-oriented information (assessed using validated instruments such as c-PEMAT-P and GQS), whereas readability refers to cognitive accessibility approximated by surface-text complexity captured by readability formulas. In patient education practice, materials are commonly expected to use plain language, minimize jargon, and present information with clear structure and actionable steps. Because widely cited grade-level readability targets are primarily defined for English texts and there is no universally accepted grade-level threshold for Chinese patient materials, the readability indices in this study are interpreted mainly as comparative benchmarks across models and topics, and are complemented by c-PEMAT-P to reflect patient-oriented understandability/actionability.

### Research questions and hypotheses

Accordingly, we formulated three operational research questions (RQs). RQ1 (model effect): Under identical prompts, do different LLMs generate patient-oriented skin cancer education materials with significantly different quality and readability? RQ2 (topic effect): Do quality and readability vary across content categories (etiology/risk, clinical manifestations, diagnosis/screening, treatment/prognosis, and prevention/patient education)? RQ3 (quality–readability relationship): Across all generated outputs, is content quality associated with readability? We hypothesized that (H1) both quality and readability would differ by model, (H2) readability (and potentially quality) would differ by content category, and (H3; central hypothesis) quality and readability would show weak or non-significant correlations, indicating partial decoupling.

Addressing this need, our study compared five prominent LLMs (Doubao, DeepSeek, Wenxin Yiyan, Tongyi Qianwen, and GPT-5), evaluating their skin cancer educational outputs using validated quality scales (C-PEMAT, GQS) and seven readability indices. Results show that while models yielded materials of comparable understandability/actionability (C-PEMAT), overall perceived quality (GQS) differed substantially; GPT-5 achieved the highest score. Crucially, readability fluctuated significantly by LLM and content category (e.g., Prevention/Treatment vs. Clinical Manifestations), revealing no single model excelled across all metrics. Furthermore, correlation analysis established that quality and readability are largely independent dimensions. This disparity—that high quality does not necessitate high readability—indicates that optimizing AI-generated health education materials requires tailored, multifaceted strategies, necessitating joint consideration of the selected LLM and the specific content topic for intended public health applications. While expert evaluations offer valuable insights, it is equally important to validate AI-generated content with end-users (patients) to ensure its clarity and relevance in real-world healthcare contexts (11).

## Materials and methods

### Ethical considerations

Data utilized in this investigation were exclusively synthetic, derived from Large Language Models (LLMs). This methodology involved no human or animal experimentation, biological samples, or personal identifying information. Consequently, ethical review is waived under established academic standards.

### Research procedure

To evaluate the performance of Large Language Models (LLMs) in dermatological knowledge dissemination, two clinical experts compiled 20 frequently asked questions (FAQs) concerning skin cancer on November 14, 2025. These questions were systematically grouped into five categories: (1) Etiology and Risk Factors, (2) Clinical Manifestations and Classification, (3) Diagnosis and Screening, (4) Treatment and Prognosis, and (5) Prevention and Patient Education. The complete set is detailed in Table 1. Researchers subsequently queried five publicly accessible LLMs—Doubao, DeepSeek, Wenxin Yiyan, Tongyi Qianwen, and GPT-5—with these FAQs. The resulting responses were analyzed across three critical metrics: readability, reliability, and quality. It must be acknowledged that using a fixed set of 20 FAQs may not fully capture the diversity of prompts and real-world contexts, thereby limiting the generalizability of the results. Furthermore, as the study was conducted in Chinese, its findings may not be entirely applicable to multilingual or cross-cultural settings. An overview of the study workflow is provided in Supplementary Figure S1.

**Table 1:** Issue list.

Issue list
1. Etiology and risk factors
1. Is long-term UV exposure the primary cause of skin cancer?
2. Does a family history of skin cancer significantly increase the risk of developing the disease?
3. Are chronic skin injuries or scars prone to inducing skin cancer?
4. Do immunocompromised individuals have a higher risk of developing skin cancer?
2. Clinical manifestations and classification
1. Does basal cell carcinoma (BCC) often present as painless nodules or plaques?
2. Does melanoma typically have uneven pigmentation and irregular borders as its typical features?
3. Is squamous cell carcinoma (SCC) prone to skin ulceration and non-healing?
4. Do skin cancer masses often be accompanied by bleeding, itching, or pain?
3. Diagnosis and screening
1. Can dermoscopy improve the diagnostic accuracy of early-stage skin cancer?
2. Is pathological tissue biopsy the sole gold standard for confirming skin cancer?
3. Should skin screening for high-risk groups be included in routine physical examinations?
4. Can PET-CT evaluate the distant metastasis of skin cancer?
4. Treatment and prognosis
1. What is the cure rate of surgical resection for early-stage skin cancer?
2. Can targeted therapy be used for patients with advanced skin cancer?
3. Does the prognosis of melanoma mainly depend on the depth of tumor invasion?
4. Can palliative radiotherapy be an option for inoperable skin cancer?
5. Prevention and patient education
1. Can proper application of SPF30 + sunscreen effectively prevent skin cancer?
2. Can wearing physical sun protection gear outdoors reduce the risk of skin cancer?
3. How can patients identify suspicious skin lesions that require urgent medical attention?
4. How often should post-operative skin cancer patients undergo re-examinations to monitor recurrence?

### LLM querying protocol and generation settings (for replication)

To strengthen reproducibility of the benchmarking procedure, we standardized the querying workflow across all five LLMs. For each of the 20 FAQs (Table 1), the same prompt template was applied to every model, and each FAQ was submitted in an independent session (i.e., a new chat/conversation was initiated for every FAQ) to avoid contextual carryover. The prompt template specified the intended audience (lay public/patients), the requested output language (Mandarin Chinese), and basic formatting requirements (e.g., plain language, structured bullet points/headings, and avoidance of unnecessary jargon) (full prompt text provided in Appendix/Supplementary material).

Where the platform allowed user control of generation parameters (e.g., temperature, top-p, maximum output length/tokens), these parameters were set identically across models; when such parameters were not exposed in the public interface, default platform settings were used and explicitly recorded. We also recorded whether web browsing/search augmentation was enabled (if applicable and user-configurable) and ensured that the same setting was applied consistently within each model. The access date (and interface/channel) for each LLM was documented to account for possible model updates over time.

The unit of analysis was one model response per FAQ (20 FAQs × 5 models = 100 outputs). Each output was exported as plain text for subsequent quality evaluation (c-PEMAT-P and GQS) and readability analysis. Complete model-specific access information and generation settings are summarized in Supplementary Table S1, and the full prompt set is provided in the Supplementary appendix to enable replication.

### Readability evaluation

Readability analysis of large language model (LLM) responses utilized multiple formulas derived from the Text Readability Assessment Tool.^1^ Acknowledging the current absence of an authoritative gold standard or consensus regarding the most reliable metric, this study consequently employs the mainstream system documented extensively in prior literature (9, 12, 13).

This study employed seven established readability metrics—the Coleman-Liau Index (CL), Linsear Write (LW), Automated Readability Index (ARI), Simple Measure of Gobbledygook (SMOG), Gunning Fog Index (GFOG), Flesch Reading Ease Score (FRES), and Flesch–Kincaid Grade Level (FKGL)—to quantify the comprehensibility and linguistic proximity of large language model (LLM)-generated output to natural, everyday speech. All outputs were generated in Mandarin Chinese and analyzed without translation. As these readability formulas were originally developed for English, we use them primarily for relative comparison of text complexity across models/topics rather than for absolute grade-level interpretation in Chinese; this is complemented by c-PEMAT-P for linguistically appropriate assessment.

The study did not conduct external factual validation by comparing the generated content with current clinical guidelines or other authoritative sources, which could have strengthened the robustness of the findings.

### Quality assessment

The assessment of literature quality and accessibility utilized two instruments: the c-PEMAT-P (Chinese version of the Patient Education Material Readability Assessment Tool) and the GQS (Global Quality Score) (1, 14–16). The c-PEMAT-P comprises 24 indicators across two dimensions: Comprehensibility (16 items, focusing on logical structure and terminology accessibility) and Practicality (8 items, evaluating specific action guidance and audience suitability). Scoring is binary (0/1), resulting in a 0–24 total score, where higher values signify greater user accessibility. For analysis, c-PEMAT-P was calculated per output as the summed 0–24 total score and then summarized as mean (±SD) across outputs within each model/category, yielding non-integer means. The GQS employs a 1–5 point scale, ranging from ‘Poor quality’ (Score 1: illogical content, no practical value) to ‘Excellent quality’ (Score 5: rigorous logic, significant practical value). Two independent clinical experts, each with over 3 years of relevant clinical experience, conducted the evaluations. Inter-rater reliability was quantified using Cohen’s Kappa (*κ*), interpreted as follows: *κ* > 0.75 indicated excellent agreement, 0.40 ≤ κ ≤ 0.75 acceptable agreement, and κ < 0.40 poor agreement. Discrepancies between the initial two raters were resolved through consensus discussions, with a third expert adjudicating the final rating when necessary. Verification confirmed that both the c-PEMAT-P and GQS scales achieved κ > 0.75, confirming excellent inter-rater reliability (17, 18). While the study did not include real-world comprehension tests or patient validation, it does not directly assess health literacy from a patient’s perspective.

### Statistical analysis

All statistical analyses were performed using IBM SPSS Statistics 25.0 (IBM Corp., Armonk, NY, USA). Continuous outcomes were summarized as mean ± standard deviation when appropriate, or as median with interquartile range for non-normally distributed variables. Normality was assessed using the Shapiro–Wilk test, and homogeneity of variance for ANOVA was assessed using Levene’s test. For outcomes meeting parametric assumptions, one-way analysis of variance (ANOVA) was used to compare groups, followed by Tukey’s honestly significant difference (HSD) test for post-hoc pairwise comparisons. For outcomes violating normality and/or homoscedasticity assumptions, the Kruskal–Wallis H test was used for multi-group comparisons, followed by Dunn’s post-hoc pairwise comparisons. Multiple comparisons were adjusted using the Bonferroni correction. Correlations between quality metrics (GQS and c-PEMAT-P) and readability indices were assessed using Pearson’s correlation coefficient (r). All tests were two-tailed, and *p* < 0.05 was considered statistically significant.

## Results

### Readability analysis

To compare the performance of five mainstream large language models (Doubek, DeepSeek, Wenxin Yiyan, Tongyi Qianwen, and GPT-5) when generating skin cancer popularization materials, we initially assessed model-level distinctions by utilizing the following metrics extracted from Table 2: quality scores (C-PEMAT and GQS) and readability indices (ARI, FRES, GFOG, FKGL, CL, SMOG, and LW).

**Table 2:** Analysis results of different large models.

Variables	Total (*n* = 100)	Deep seek (*n* = 20)	Doubao (*n* = 20)	GPT-5 (*n* = 20)	Tongyi Qianwen (*n* = 20)	Wenxin Yiyan (*n* = 20)	Statistic	*P*
C-PEMAT score, Mean ± SD	8.92 ± 0.86	8.80 ± 0.77	8.75 ± 1.21	9.40 ± 0.50	8.95 ± 0.83	8.70 ± 0.73	*F* = 2.29	0.065
GQS score, Mean ± SD	3.09 ± 1.08	3.30 ± 0.57	3.45 ± 0.60	4.40 ± 0.68	2.20 ± 0.83	2.10 ± 0.64	F = 40.50	**<.001**
ARI, M (Q₁, Q₃)	16.67 (14.86, 18.17)	16.14 (13.76, 18.23)	16.91 (16.20, 18.09)	18.22 (16.35, 19.12)	17.00 (15.55, 18.17)	14.72 (13.23, 16.35)	χ^2^ = 17.82#	**0.001**
FRES, M (Q₁, Q₃)	26.00 (13.00, 37.50)	30.50 (20.75, 39.25)	23.00 (12.75, 34.75)	21.00 (9.75, 31.00)	15.00 (7.75, 35.00)	38.50 (25.75, 44.75)	χ^2^ = 16.52#	**0.002**
GFOG, M (Q₁, Q₃)	15.20 (13.97, 17.50)	15.15 (12.78, 16.70)	15.85 (14.67, 16.60)	15.95 (14.20, 18.15)	16.65 (14.52, 18.07)	13.75 (12.33, 15.03)	χ^2^ = 11.89#	**0.018**
FKGL, M (Q₁, Q₃)	15.00 (12.95, 16.57)	14.96 (13.42, 16.68)	14.96 (13.53, 16.39)	16.07 (14.85, 17.63)	15.20 (13.24, 16.91)	13.29 (12.01, 15.64)	χ^2^ = 11.09#	**0.026**
CL, M (Q₁, Q₃)	15.53 (13.78, 17.78)	14.15 (12.53, 16.16)	16.19 (13.85, 17.43)	16.82 (15.07, 18.12)	18.23 (15.43, 19.04)	13.59 (12.40, 14.83)	χ^2^ = 28.32#	**<.001**
SMOG, M (Q₁, Q₃)	13.32 (12.22, 14.57)	13.32 (11.98, 14.84)	13.28 (12.61, 14.11)	14.36 (13.47, 15.18)	13.02 (12.20, 14.14)	12.32 (10.96, 13.69)	χ^2^ = 13.02#	**0.011**
LW, M (Q₁, Q₃)	60.00 (56.75, 64.00)	62.00 (58.50, 64.50)	57.00 (54.00, 61.25)	58.50 (57.00, 61.00)	60.00 (56.75, 64.25)	62.50 (58.00, 64.75)	χ^2^ = 7.74#	0.102

F, ANOVA, #: Kruskal-waills test. SD: standard deviation, M: Median, Q₁: 1st Quartile, Q₃: 3st Quartile.Bold values indicate statistically significant results (*p* < 0.05).

At the model level, GQS scores exhibited significant variation (*F* = 40.50, *p* < 0.001). GPT-5 recorded the highest mean GQS score (4.40), underscoring its superior overall content quality compared to lower scores registered by Doubao (3.45), DeepSeek (3.30), Tongyi Qianwen (2.20), and Wenxin Yiyan (~2.10). Conversely, C-PEMAT scores did not differ significantly across models ($*p* = 0.065$), as evidenced by mean values clustered within a restricted range (8.70–9.40), suggesting broad equivalence in understandability and actionability across the five models.

Readability metrics demonstrated greater heterogeneity across models. Six indices showed significant inter-model variation: ARI (GPT-5 median: 18.22 vs. DeepSeek: 16.14; χ^2^ = 17.82, *p* = 0.001), FRES (χ^2^ = 16.52, *p* = 0.002), CL (χ^2^ = 28.32, *p* < 0.001), SMOG (χ^2^ = 13.02, *p* = 0.011), GFOG (χ^2^ = 11.89, *p* = 0.018), and FKGL (χ^2^ = 11.09, *p* = 0.026). The LW index, however, failed to reach statistical significance (*p* = 0.102). Collectively, these findings confirm that the readability of skin cancer educational content is model-dependent, though no single model demonstrated consistent superiority across all indices. Post-hoc pairwise comparisons were conducted using Dunn’s test with Bonferroni correction; detailed pairwise Z statistics and adjusted *p* values are provided in Supplementary Table S2.

Analysis of quality and readability by content category (Table 3) revealed a significant divergence in C-PEMAT scores (*F* = 3.86, *p* = 0.006). The Prevention and Treatment category recorded the highest mean score (9.50), considerably exceeding the lower mean (8.65) of the Clinical Manifestation category. This disparity suggests model-generated prevention-and treatment-related content was inherently more understandable and actionable. Conversely, GQS scores exhibited no significant difference across content categories (*p* = 0.336).

**Table 3:** Analysis results by content category.

Variables	Total (*n* = 100)	Clinical manifestations and classification (*n* = 20)	Diagnosis and screening (*n* = 20)	Etiology and risk factors (*n* = 20)	Prevention and patient education (n = 20)	Treatment and prognosis (*n* = 20)	Statistic	*P*
C-PEMAT score, Mean ± SD	8.92 ± 0.86	8.65 ± 0.88	8.90 ± 0.85	8.95 ± 0.94	9.50 ± 0.51	8.60 ± 0.82	*F* = 3.86	**0.006**
GQS score, Mean ± SD	3.09 ± 1.08	3.10 ± 1.21	2.85 ± 0.93	3.10 ± 1.07	3.15 ± 1.04	3.25 ± 1.21	*F* = 0.36	0.836
ARI, M (Q₁, Q₃)	16.67 (14.86, 18.17)	13.66 (13.03, 15.62)	17.77 (16.73, 18.84)	17.23 (16.14, 18.04)	16.75 (14.92, 18.00)	16.21 (15.18, 18.33)	χ^2^ = 18.19#	**0.001**
FRES, M (Q₁, Q₃)	26.00 (13.00, 37.50)	38.00 (25.50, 44.25)	13.00 (8.50, 21.00)	18.50 (13.00, 26.75)	36.50 (29.25, 45.75)	27.50 (9.00, 34.25)	χ^2^ = 32.88#	**<.001**
GFOG, M (Q₁, Q₃)	15.20 (13.97, 17.50)	12.80 (11.78, 14.53)	17.40 (15.40, 18.47)	16.60 (15.88, 18.07)	14.15 (12.80, 14.62)	15.80 (14.67, 17.80)	χ^2^ = 42.51#	**<.001**
FKGL, M (Q₁, Q₃)	15.00 (12.95, 16.57)	12.59 (11.62, 14.40)	16.52 (15.86, 17.85)	15.71 (14.77, 16.50)	13.82 (12.12, 15.23)	14.79 (13.60, 16.66)	χ^2^ = 25.88#	**<.001**
CL, M (Q₁, Q₃)	15.53 (13.78, 17.78)	13.82 (12.47, 14.80)	17.24 (15.73, 18.32)	16.62 (15.47, 18.23)	13.94 (12.67, 16.14)	15.48 (13.32, 18.24)	χ^2^ = 21.92#	**<.001**
SMOG, M (Q₁, Q₃)	13.32 (12.22, 14.57)	11.34 (10.60, 12.26)	14.11 (13.70, 14.84)	13.96 (13.07, 14.81)	12.44 (11.69, 13.47)	13.28 (12.68, 14.43)	χ^2^ = 31.84#	**<.001**
LW, M (Q₁, Q₃)	60.00 (56.75, 64.00)	65.00 (60.00, 68.00)	56.00 (53.00, 61.00)	60.50 (59.00, 64.00)	61.50 (58.50, 66.50)	56.50 (53.00, 58.00)	χ^2^ = 27.19#	**<.001**

F, ANOVA; #, Kruskal-waills test.Bold values indicate statistically significant results (*p* < 0.05).

Readability indices, however, varied markedly across content categories, exhibiting significant differences across all tested metrics: ARI (χ^2^ = 18.19, *p* = 0.001), FRES (χ^2^ = 32.88, *p* < 0.001), GFOG (χ^2^ = 42.51, p < 0.001), FKGL (χ^2^ = 25.88, p < 0.001), CL (χ^2^ = 21.92, p < 0.001), SMOG (χ^2^ = 31.84, p < 0.001), and LW (χ^2^ = 27.19, p < 0.001). The Prevention and Treatment category, overall, demonstrated the most favorable performance across several of these metrics.

These findings demonstrate that GPT-5 achieves the highest overall Content Quality Score (GQS) in skin cancer science popularization. Conversely, readability depends significantly on both model choice and content category. Consequently, optimizing educational materials for specific applications necessitates the joint consideration of model selection and content tailoring.

Because these readability formulas were originally developed for English and there is no universally accepted grade-level threshold for Chinese patient materials, we interpret the indices primarily for relative comparisons across models and content categories. For transparency and comparability with prior English-language health communication literature, we additionally summarized the proportion of outputs with FKGL ≤ 8 (and ≤ 6) as an exploratory analysis; detailed proportions are reported in Supplementary Table S4, S5.

### Quality analysis

**Figure 1:**
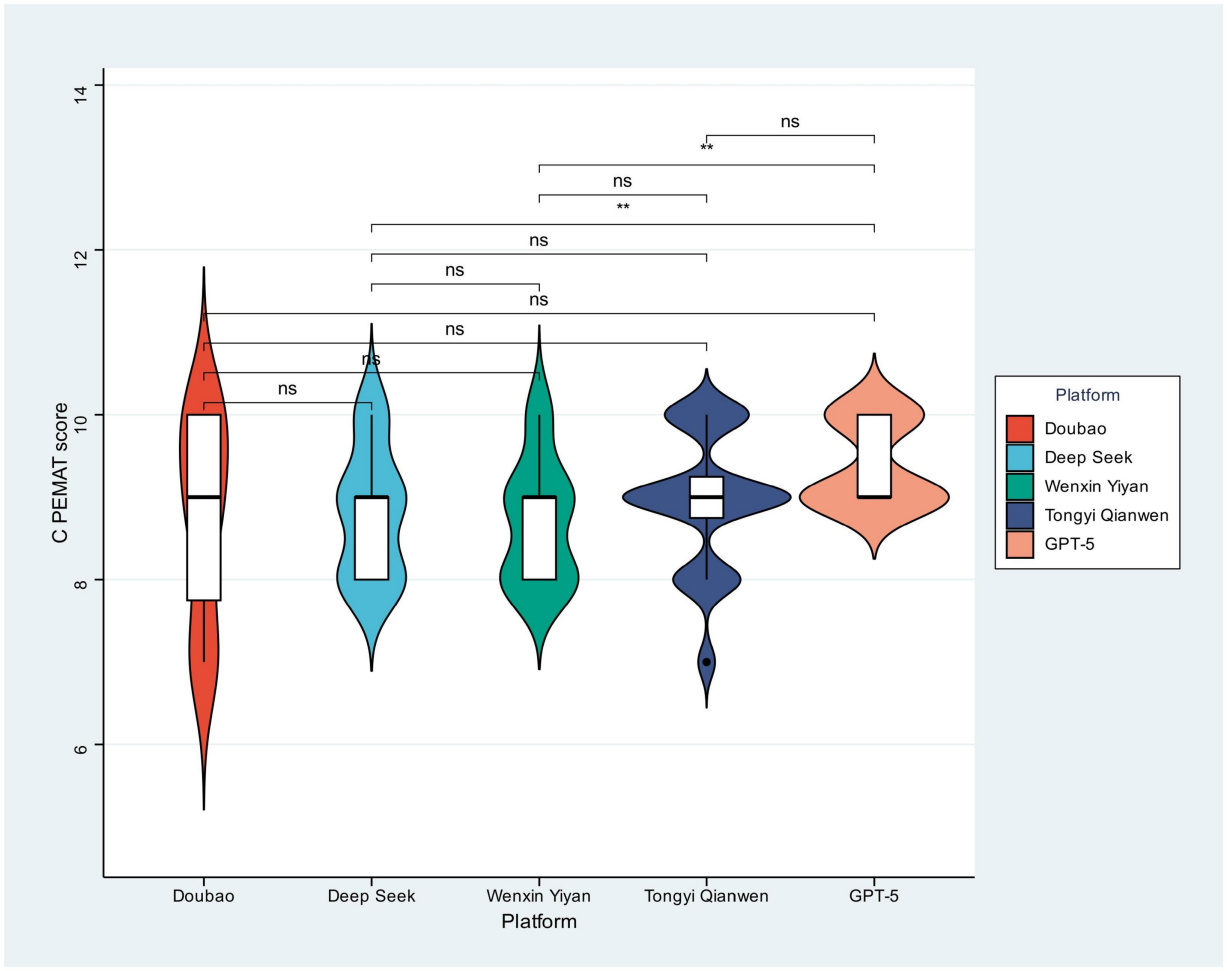
c-PEMAT-P scores of different large models.

The C-PEMAT score distributions across the five models were visualized (Figure 1) to assess differences in the educational content’s understandability and actionability. While visual inspection suggested variation in distribution shape and density—GPT-5 scores, for instance, appeared more concentrated at higher values—all pairwise comparisons proved statistically non-significant (“ns”). This outcome indicates that the C-PEMAT quality of educational materials generated by these large language models (LLMs) was broadly similar, demonstrating no statistically demonstrable advantage for any single model.

**Figure 2:**
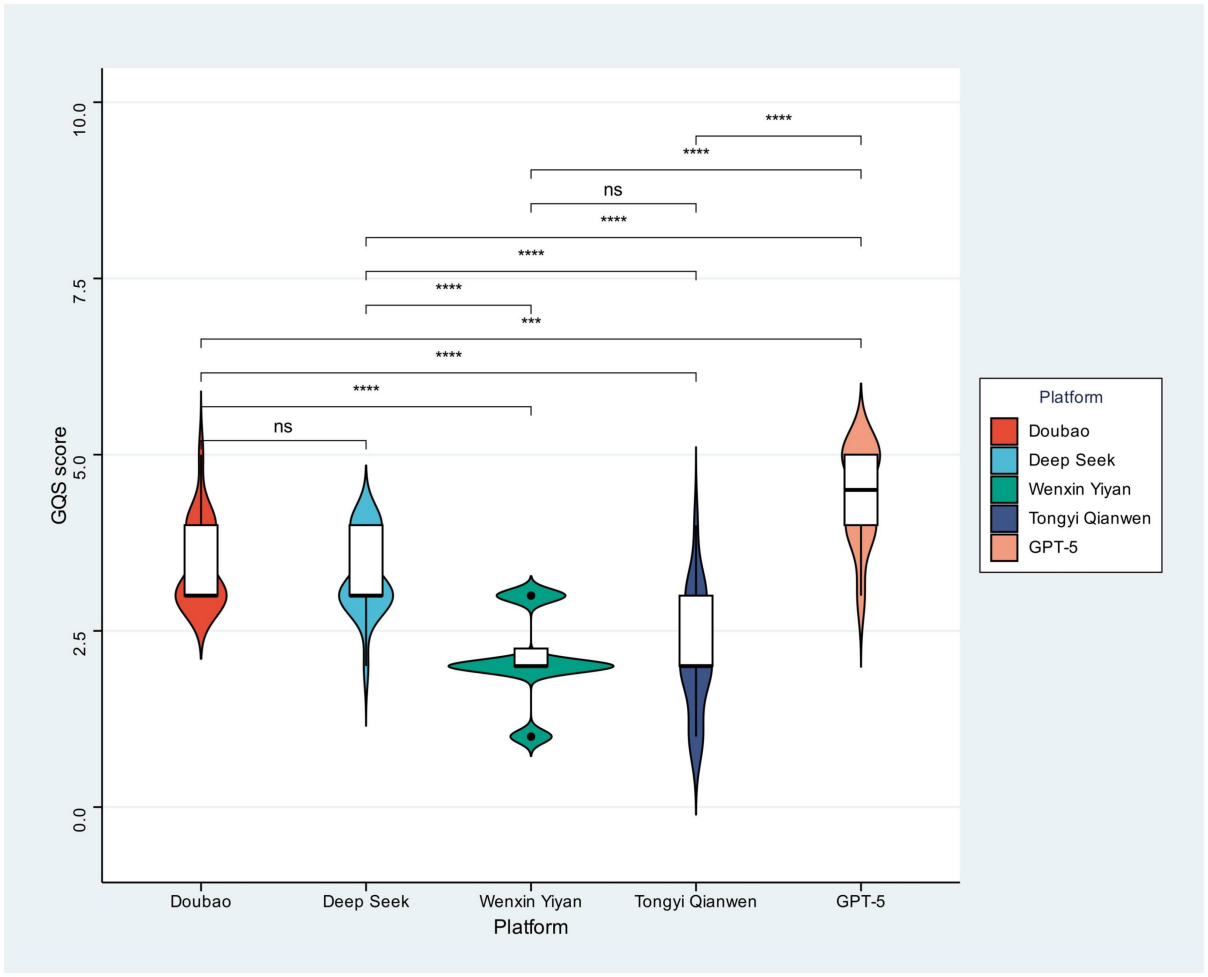
GQS scores of different large models.

Figure 2 illustrates the distribution of Global Quality Scale (GQS) scores, which reveal statistically significant disparities across the five models, a stark contrast to C-PEMAT results. Specifically, GPT-5 registered the highest median GQS score, correlating with a demonstrable upward shift in its distribution. Subsequent pairwise analyses confirmed GPT-5’s consistent superior ranking over all tested competitors—Doubao, DeepSeek, Wenxin Yiyan, and Tongyi Qianwen (all statistically significant, labeled “****”)—whereas Wenxin Yiyan consistently exhibited comparatively lower scores. These findings collectively establish GPT-5’s superior overall perceived quality (GQS) for skin cancer educational content.

### Correlation analysis

**Figure 3:**
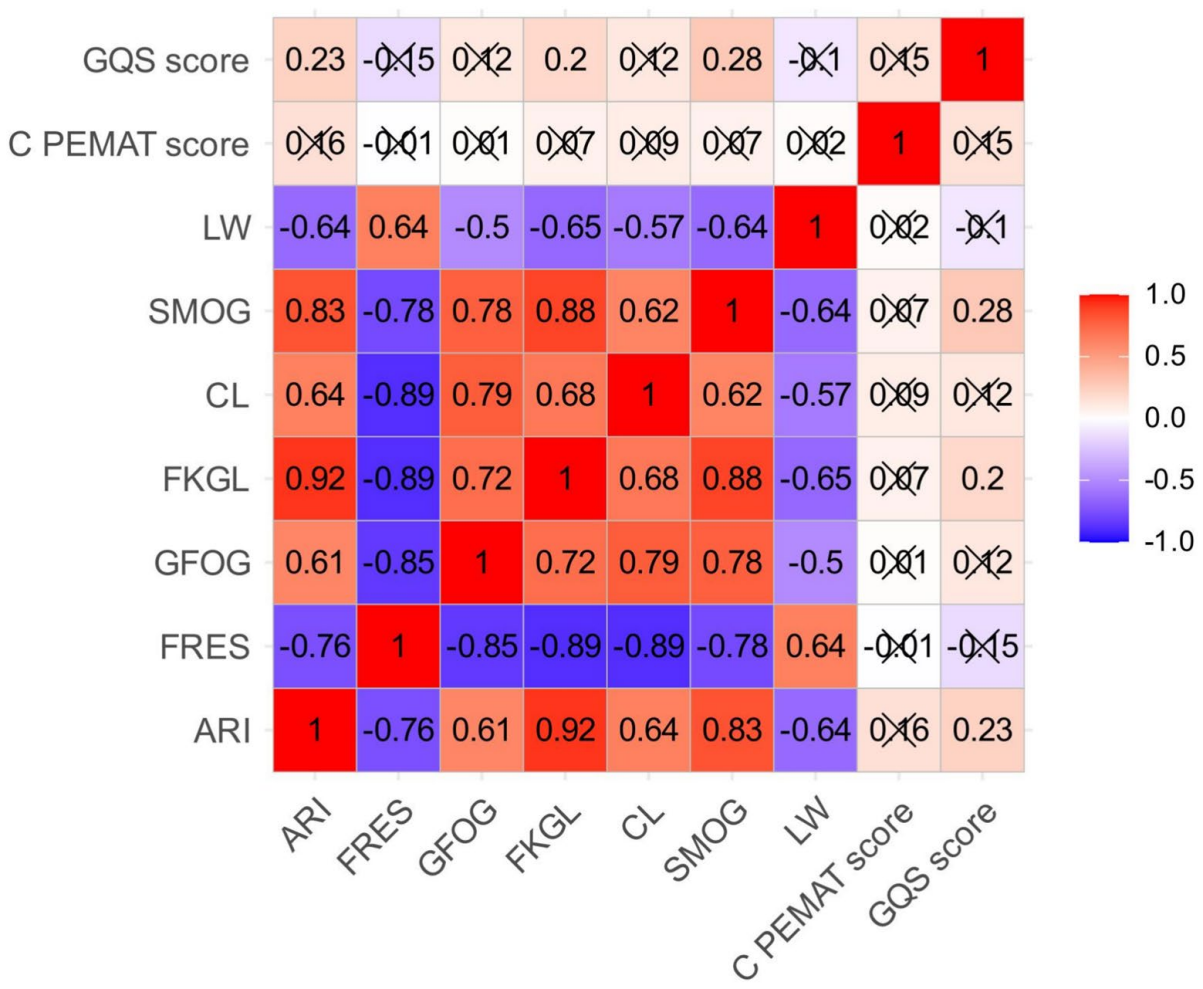
Heatmap of correlations among different indicators.

Correlation analyses were conducted using Pearson’s correlation coefficient (r) across all outputs (*n* = 100). The full correlation matrix (r) with two-tailed *p* values is provided in Supplementary Table S3, and the heatmap is shown in Figure 3. A correlation heatmap was employed to analyze the correlation structure between quality assessments and textual readability, specifically comparing the two quality metrics (GQS and C-PEMAT) against seven readability indices (ARI, FRES, GFOG, FKGL, CL, SMOG, and LW) across all skin cancer educational texts.

The analysis revealed a strong positive correlation between GQS and C-PEMAT, demonstrating consistency in ranking overall content quality. Substantial shared variance in capturing textual complexity was similarly evident across several readability indices, where strong positive correlations emerged between SMOG and CL, GFOG and FKGL, and ARI and FRES.

By contrast, GQS and C-PEMAT demonstrated only weak, often negative, correlations with most readability indices; the associations were weakest with LW. The weak correlation between content quality (GQS/C-PEMAT) and readability indices suggests that these two factors function independently. While high-quality content can be comprehensive and accurate, it may not always meet simplified readability requirements, indicating that improving one aspect does not necessarily lead to improvement in the other. Consequently, optimizing AI-generated skin cancer educational materials requires complementary strategies that target these elements separately, as high quality does not correlate with inherent readability.

## Discussion

Large Language Models (LLMs) yield skin cancer education content of high quality; however, textual readability proves highly heterogeneous across models and topics, being only weakly correlated with content quality. Consequently, selecting and configuring LLMs for patient education mandates explicit consideration of both content quality and readability, as improvements in one dimension do not automatically translate to the other.

The long-standing observation that conventional skin cancer patient information often exceeds internationally recommended reading levels, even from professional organizations (19–21), motivated this study. Prior analyses of online resources consistently report median reading levels spanning tenth to twelfth grade (19–21), rendering materials inaccessible to the average reader despite the critical need for clarity in prevention and treatment decision-making. We therefore investigated whether contemporary Large Language Models (LLMs) could overcome these accessibility limitations while simultaneously maintaining informational quality. Results indicated GPT-5 achieved the highest Global Quality Scale (GQS) score, significantly outperforming Doubao, DeepSeek, Tongyi Qianwen, and Wenxin Yiyan, whereas C-PEMAT scores showed no statistically significant inter-model differences. This suggests GPT-5 provides superior comprehensiveness and currency, while all five models generate similarly understandable and actionable content when assessed using the validated Chinese version of the Patient Education Materials Assessment Tool (C-PEMAT) (1, 22). These findings complement prior work demonstrating that LLMs match or exceed the quality of traditional oncology and dermatology patient resources (6–8), simultaneously underscoring significant non-interchangeability among models.

Our second contribution involves comparing readability systematically across models using seven established indices. Unlike the convergent C-PEMAT scores, these indices revealed pronounced cross-model variability: no single system emerged as best-performing across measures including the Automated Readability Index, Flesch–Kincaid Grade Level, Gunning Fog, SMOG, Coleman–Liau, Linsear Write, and Chinese-specific metrics. This parallels large-scale analyses showing that different readability formulas agree only moderately, and that most conventional health materials persistently exceed the recommended sixth- to eighth-grade level (1, 19, 20). These findings also extend recent LLM-focused studies in dermatology and other specialties, where ChatGPT-generated handouts offered marginal readability improvements over legacy patient education materials but failed to reach the target level consistently (5, 8, 22, 23). Crucially, by exposing substantial cross-model heterogeneity on identical prompts, our results underscore that model choice alone dictates the reading burden patients face, even under ostensibly similar generation instructions.

Thirdly, quality and readability strongly depend on the type of skin cancer content generated. Specifically, texts focusing on prevention and treatment achieved higher C-PEMAT scores and more favorable readability values than sections describing clinical manifestations, whereas GQS scores remained statistically uniform across content categories. This aligns with earlier work on internet-based skin cancer resources, where campaign-driven prevention and treatment pages were modestly easier to read than pathology- or prognosis-focused pages, though they still remained above recommended levels (19, 24). Our results suggest that large language models inherit — and may even amplify—this imbalance: broad prompting leads models to prioritize mechanistic and diagnostic details that increase lexical and syntactic complexity, particularly in sections on clinical features. Crucially, the relatively uniform GQS scores across categories indicate expert perception of stable medical accuracy and usefulness. Consequently, these patterns reinforce the need for deliberate prompt engineering or post-processing when generating education materials specifically aimed at symptom recognition and early presentation.

The study’s most conceptually significant finding is the decoupling of content quality from readability. A strong positive correlation between GQS and C-PEMAT demonstrated that comprehensive and reliable materials were perceived by expert raters as more understandable and actionable, a result consistent with prior PEMAT-based evaluations (8, 19, 22). Conversely, these quality metrics showed only weak, predominantly negative correlations with most readability indices. That is, greater perceived quality and patient-friendliness were not systematically associated with simplified surface text structure or reduced grade-level scores. This finding refutes the common assumption within the LLM and health communication literature that optimizing readability metrics necessarily advances health literacy. For example, studies focusing on optimizing Flesch–Kincaid or SMOG scores via ChatGPT re-writing reported numerical gains but failed to assess the accuracy and actionability of key messages directly (5). Similarly, recent blinded comparisons of multiple LLMs generating dermatology handouts have emphasized mean readability and PEMAT scores but omitted formal modeling of their interrelationship (6, 22). By jointly quantifying these dimensions in a single framework, our data strongly suggest that readability formulas capture only a narrow slice of the factors governing the genuine usability of LLM-generated content for patients.

Our results validate and nuance the existing literature positioning large language models (LLMs) as promising patient education tools. Scoping reviews and empirical evaluations routinely showcase LLMs’ flexibility, scalability, and high face validity across oncology and dermatology. Yet, these studies also raise concerns regarding hallucinations, lack of personalization, and potential biases (6, 8, 22, 23). Building upon this foundation, our study offers a focused examination in skin cancer education that foregrounds the critical tension between quality and readability, rather than treating these metrics as interchangeable surrogates. Crucially, the finding that GPT-5 yielded the highest Global Quality Score (GQS) but failed to universally dominate readability suggests that future evaluations must report both outcomes and consider task-specific trade-offs—e.g., maximizing accuracy for complex treatment discussions while allowing aggressive simplification for primary prevention messaging.

This study presents several critical limitations. First, our scope was narrow: the analysis restricted itself to a single disease area, a fixed set of standardized prompts, and content categories. Consequently, the observed patterns—specifically the decoupling of quality and readability—may not generalize across other diseases, languages, or cultural contexts. Second, we relied on expert ratings (GQS and C-PEMAT) as proxies for patient-centered outcomes; while these instruments are widely validated, they fail to directly capture patients’ comprehension, trust, or resultant behavioral change. Third, readability was assessed via automated indices applied solely to single model outputs, thereby ignoring potential variability stemming from differing sampling temperatures, prompt refinements, or iterative clinical co-creation. Fourth, the systematic assessment of factual accuracy, hallucination rates, or harmful content was limited to the implicit GQS ratings. Critically, LLM outputs were not compared with contemporaneous external resources, such as web pages or professionally authored pamphlets. Fifth, this study did not include a subgroup analysis based on education levels, limiting our ability to assess the practical relevance of the materials for low-literacy populations. Sixth, this study provides valuable insights into the quality and readability of AI-generated health content, the findings are based on expert assessments rather than direct feedback from patients. Finally, given the rapid evolution of the commercial LLM landscape, our snapshot of five systems is inherently fleeting, risking rapid obsolescence as architectures and safety layers are updated.

Addressing these limitations requires several future research avenues. First, we must expand this approach to multiple diseases, languages, and health-care settings, simultaneously integrating both expert- and patient-level outcomes, including comprehension tests, decisional conflict, and behaviorally anchored measures of health literacy. Second, prospective trials should rigorously compare pipelines that leverage state-of-the-art models (e.g., GPT-5) for initial content generation, followed by algorithmic or interactive rewriting to target specific reading levels, while explicitly monitoring for C-PEMAT and GQS score degradation. Third, collaborations among clinicians, health communication specialists, and AI developers must define domain-specific prompt templates, guardrails, and evaluation dashboards that simultaneously track readability, quality, and safety. Third, Future research should focus on incorporating patient-centered validation to refine AI-generated educational materials and ensure their relevance in real-world healthcare settings. Finally, at a systems level, embedding LLM-generated but clinician-reviewed skin cancer education within electronic health records and teledermatology platforms could close long-standing gaps in patient information, thereby ensuring accountability. Ultimately, by treating quality and readability as complementary rather than interchangeable design targets, our work establishes the foundation for safer and more equitable deployment of large language models in skin cancer education and patient education more broadly.

## Data Availability

The raw data supporting the conclusions of this article will be made available by the authors, without undue reservation.

## Footnotes

1
http://readabilityformulas.com/

## References

[1] Shan Y Ji M Dong Z Xing Z Wang D Cao X. The Chinese version of the patient education materials assessment tool for printable materials: translation, adaptation, and validation study. J Med Internet Res. (2023) 25:e39808. doi: 10.2196/39808, 37200085 PMC10236277

[2] Nadratowski A Shoots-Reinhard B Shafer A Detweiler-Bedell J Detweiler-Bedell B Leachman S . Evidence-based communication to increase melanoma knowledge and skin checks. JID Innov. (2023) 4:100253. doi: 10.1016/j.xjidi.2023.100253 38328593 PMC10847376

[3] Jawad R Jawad R. Comparison feed forward back propagation networks (FFBPNs) with support vector machine (SVM) for diagnosis skin cancer based on images. Vokasi Unesa Bull Eng Technol Appl Sci. (2025) 2:127–35. doi: 10.26740/vubeta.v2i2.36117

[4] Mac O Ayre J Bell K McCaffery K Muscat DM. Comparison of readability scores for written health information across formulas using automated vs manual measures. JAMA Netw Open. (2022) 5:e2246051. doi: 10.1001/jamanetworkopen.2022.46051, 36508219 PMC9856555

[5] Aydin S Karabacak M Vlachos V Margetis K. Large language models in patient education: a scoping review of applications in medicine. Front Med. (2024) 11:1477898. doi: 10.3389/fmed.2024.1477898, 39534227 PMC11554522

[6] Lambert R Choo Z Gradwohl K Choo Z-Y Schroedl L Ruiz De Luzuriaga A. Assessing the application of large language models in generating dermatologic patient education materials according to reading level: qualitative study. JMIR Dermatol. (2024) 7:e55898. doi: 10.2196/55898, 38754096 PMC11140271

[7] Roster K Kann RB Farabi B Gronbeck C Brownstone N Lipner SR. Readability and health literacy scores for ChatGPT-generated dermatology public education materials: cross-sectional analysis of sunscreen and melanoma questions. JMIR Dermatol. (2024) 7:e50163. doi: 10.2196/50163, 38446502 PMC10955394

[8] Chang CT Ticknor IL Spinelli J Spinelli J-A Bhatia BK Marwaha S . Comparison of large language models in generating patient handouts for the dermatology clinic: a blinded study. JAAD Int. (2024) 15:152–4. doi: 10.1016/j.jdin.2024.02.010, 38571697 PMC10988028

[9] Steeb T Reinhardt L Harla M Ting PS Trivedi H Vipani A . Assessment of the quality, understandability, and reliability of YouTube videos as a source of information on basal cell carcinoma: web-based analysis. JMIR Cancer. (2022) 8:e29581. doi: 10.3350/cmh.2023.0089 35275067 PMC8956995

[10] Lee K Yee D Peterson H Huang MY Kingston P Agüero R . Readability, quality, and comprehensiveness of online health resources for skin cancer in skin of color. Int J Dermatol. (2023) 62:e532–4. doi: 10.1111/ijd.16674, 37039526

[11] Hamam H. Rethinking intelligence: from human cognition to artificial futures. Vokasi Unesa Bull Eng Technol Appl Sci. (2025) 2:531–48. doi: 10.26740/vubeta.v2i3.44232

[12] Mondal H Mondal S. ChatGPT in academic writing: maximizing its benefits and minimizing the risks. Indian J Ophthalmol. (2023) 71:3600–6. doi: 10.4103/ijo.ijo, 37991290 PMC10788737

[13] Hamnes B van Eijk-Hustings Y Primdahl J. Readability of patient information and consent documents in rheumatological studies. BMC Med Ethics. (2016) 17:42. doi: 10.1186/s12910-016-0126-0, 27422433 PMC4947296

[14] Barlas T Ecem Avci D Cinici B Ozkilicaslan H Muhittin Yalcin M Eroglu Altinova A. The quality and reliability analysis of YouTube videos about insulin resistance. Int J Med Inform. (2023) 170:104960. doi: 10.1016/j.ijmedinf.2022.104960, 36525801

[15] Sun F Zheng S Wu J. Quality of information in gallstone disease videos on TikTok: cross-sectional study. J Med Internet Res. (2023) 25:e39162. doi: 10.2196/39162, 36753307 PMC9947761

[16] Subramanian T Araghi K Akosman I Tuma O Hassan A Lahooti A . Quality of spine surgery information on social media: a DISCERN analysis of TikTok videos. Neurospine. (2023) 20:1443–9. doi: 10.14245/ns.2346700.350, 38171310 PMC10762400

[17] Lin I Shen Y Shih M Lin I-T Shen Y-M Shih M-J . Short video addiction on the interaction of creative self-efficacy and career interest to innovative design profession students. Healthcare. (2023) 11:579. doi: 10.3390/healthcare11040579, 36833113 PMC9956146

[18] Wang L Li Y Gu J Xiao L. A quality analysis of thyroid cancer videos available on TikTok. Front Public Health. (2023) 11:1049728. doi: 10.3389/fpubh.2023.1049728 37033054 PMC10076716

[19] Basch CH Fera J Ethan D Garcia P Perin D Basch CE. Readability of online material related to skin cancer. Public Health. (2018) 163:137–40. doi: 10.1016/j.puhe.2018.07.009 30149263

[20] John AM John ES Hansberry DR Lambert WC. Assessment of online patient education materials from major dermatologic associations. J Clin Aesthet Dermatol. (2016) 9:23–8. PMC5110326 27878059

[21] Steeb T Reinhardt L Harla M Heppt MV Meier F Berking C. Assessment of the quality, understandability, and reliability of YouTube videos as a source of information on basal cell carcinoma: web-based analysis. JMIR Cancer. (2022) 8:e29581. doi: 10.2196/29581 35275067 PMC8956995

[22] Furukawa E Okuhara T Liu M Okada H Kiuchi T. Evaluating online and offline health information with the patient education materials assessment tool: protocol for a systematic review. JMIR Res Protoc. (2025) 14:e63489. doi: 10.2196/63489, 39813665 PMC11780281

[23] Swisher AR Wu AW Liu GC Lee MK Carle TR Tang DM. Enhancing health literacy: evaluating the readability of patient handouts revised by ChatGPT's large language model. Otolaryngol Head Neck Surg. (2024) 171:1751–7. doi: 10.1002/ohn.927, 39105460

[24] Goorman E Mittal S Choi JN. Assessing readability of skin cancer screening resources: a comparison of online websites and ChatGPT responses. J Cancer Educ. (2025) 2025:10–1007. doi: 10.1007/s13187-025-02683-2 PMC13222313 40591107

