# Supplementary material for: Decoupled quality and readability in skin cancer education from large language models
Source: Front Public Health. 2026 Feb 20;14:1777577. doi: 10.3389/fpubh.2026.1777577 (PMC12962940; doi:10.3389/fpubh.2026.1777577)
Supplement: Supplementary file 2 [file Table_2.docx]

**Supplementary Tables (S2–S5)**

S2 reports Dunn’s post-hoc pairwise comparisons (Z statistics) following Kruskal–Wallis tests, with Bonferroni-adjusted P values. S3 reports Pearson correlation coefficients (r) across all outputs (n = 100) with two-tailed and Bonferroni-adjusted P values. S4,S5 summarizes exploratory readability-cutoff proportions (FKGL and SMOG) by model and by content category.

**Supplementary Table S2. Dunn’s post-hoc pairwise comparisons (Bonferroni-adjusted)**

One row per pairwise comparison; grouping factor indicates whether comparisons are across models or content categories.

| **Outcome / metric** | **Grouping factor** | **Comparison (A vs B)** | **Test statistic (Z)** | **Adjusted P value (Bonferroni)** | **Significant (Y/N)** |
| --- | --- | --- | --- | --- | --- |
| ARI | Model | Deep Seek vs Doubao | -1.079 | 1.0000 | N |
| ARI | Model | Deep Seek vs GPT-5 | -1.809 | 0.7038 | N |
| ARI | Model | Deep Seek vs Tongyi Qianwen | -0.605 | 1.0000 | N |
| ARI | Model | Deep Seek vs Wenxin Yiyan | 2.117 | 0.3423 | N |
| ARI | Model | Doubao vs GPT-5 | -0.730 | 1.0000 | N |
| ARI | Model | Doubao vs Tongyi Qianwen | 0.474 | 1.0000 | N |
| ARI | Model | Doubao vs Wenxin Yiyan | 3.197 | 0.0139 | Y |
| ARI | Model | GPT-5 vs Tongyi Qianwen | 1.204 | 1.0000 | N |
| ARI | Model | GPT-5 vs Wenxin Yiyan | 3.927 | 8.607e-04 | Y |
| ARI | Model | Tongyi Qianwen vs Wenxin Yiyan | 2.722 | 0.0648 | N |
| ARI | Content category | Clinical Manifestations and Classification vs Diagnosis and Screening | -4.186 | 2.843e-04 | Y |
| ARI | Content category | Clinical Manifestations and Classification vs Etiology and Risk Factors | -2.788 | 0.0531 | N |
| ARI | Content category | Clinical Manifestations and Classification vs Prevention and Patient Education | -2.196 | 0.2806 | N |
| ARI | Content category | Clinical Manifestations and Classification vs Treatment and Prognosis | -2.289 | 0.2208 | N |
| ARI | Content category | Diagnosis and Screening vs Etiology and Risk Factors | 1.398 | 1.0000 | N |
| ARI | Content category | Diagnosis and Screening vs Prevention and Patient Education | 1.989 | 0.4667 | N |
| ARI | Content category | Diagnosis and Screening vs Treatment and Prognosis | 1.897 | 0.5787 | N |
| ARI | Content category | Etiology and Risk Factors vs Prevention and Patient Education | 0.591 | 1.0000 | N |
| ARI | Content category | Etiology and Risk Factors vs Treatment and Prognosis | 0.499 | 1.0000 | N |
| ARI | Content category | Prevention and Patient Education vs Treatment and Prognosis | -0.093 | 1.0000 | N |
| FRES | Model | Deep Seek vs Doubao | 0.951 | 1.0000 | N |
| FRES | Model | Deep Seek vs GPT-5 | 2.083 | 0.3727 | N |
| FRES | Model | Deep Seek vs Tongyi Qianwen | 1.693 | 0.9046 | N |
| FRES | Model | Deep Seek vs Wenxin Yiyan | -1.497 | 1.0000 | N |
| FRES | Model | Doubao vs GPT-5 | 1.131 | 1.0000 | N |
| FRES | Model | Doubao vs Tongyi Qianwen | 0.742 | 1.0000 | N |
| FRES | Model | Doubao vs Wenxin Yiyan | -2.448 | 0.1436 | N |
| FRES | Model | GPT-5 vs Tongyi Qianwen | -0.390 | 1.0000 | N |
| FRES | Model | GPT-5 vs Wenxin Yiyan | -3.580 | 0.0034 | Y |
| FRES | Model | Tongyi Qianwen vs Wenxin Yiyan | -3.190 | 0.0142 | Y |
| FRES | Content category | Clinical Manifestations and Classification vs Diagnosis and Screening | 4.373 | 1.226e-04 | Y |
| FRES | Content category | Clinical Manifestations and Classification vs Etiology and Risk Factors | 3.042 | 0.0235 | Y |
| FRES | Content category | Clinical Manifestations and Classification vs Prevention and Patient Education | -0.393 | 1.0000 | N |
| FRES | Content category | Clinical Manifestations and Classification vs Treatment and Prognosis | 2.301 | 0.2140 | N |
| FRES | Content category | Diagnosis and Screening vs Etiology and Risk Factors | -1.330 | 1.0000 | N |
| FRES | Content category | Diagnosis and Screening vs Prevention and Patient Education | -4.765 | 1.884e-05 | Y |
| FRES | Content category | Diagnosis and Screening vs Treatment and Prognosis | -2.072 | 0.3827 | N |
| FRES | Content category | Etiology and Risk Factors vs Prevention and Patient Education | -3.435 | 0.0059 | Y |
| FRES | Content category | Etiology and Risk Factors vs Treatment and Prognosis | -0.742 | 1.0000 | N |
| FRES | Content category | Prevention and Patient Education vs Treatment and Prognosis | 2.694 | 0.0707 | N |
| GFOG | Model | Deep Seek vs Doubao | -0.929 | 1.0000 | N |
| GFOG | Model | Deep Seek vs GPT-5 | -1.183 | 1.0000 | N |
| GFOG | Model | Deep Seek vs Tongyi Qianwen | -1.521 | 1.0000 | N |
| GFOG | Model | Deep Seek vs Wenxin Yiyan | 1.507 | 1.0000 | N |
| GFOG | Model | Doubao vs GPT-5 | -0.253 | 1.0000 | N |
| GFOG | Model | Doubao vs Tongyi Qianwen | -0.591 | 1.0000 | N |
| GFOG | Model | Doubao vs Wenxin Yiyan | 2.437 | 0.1482 | N |
| GFOG | Model | GPT-5 vs Tongyi Qianwen | -0.338 | 1.0000 | N |
| GFOG | Model | GPT-5 vs Wenxin Yiyan | 2.690 | 0.0714 | N |
| GFOG | Model | Tongyi Qianwen vs Wenxin Yiyan | 3.028 | 0.0246 | Y |
| GFOG | Content category | Clinical Manifestations and Classification vs Diagnosis and Screening | -4.737 | 2.166e-05 | Y |
| GFOG | Content category | Clinical Manifestations and Classification vs Etiology and Risk Factors | -4.568 | 4.916e-05 | Y |
| GFOG | Content category | Clinical Manifestations and Classification vs Prevention and Patient Education | -0.327 | 1.0000 | N |
| GFOG | Content category | Clinical Manifestations and Classification vs Treatment and Prognosis | -3.546 | 0.0039 | Y |
| GFOG | Content category | Diagnosis and Screening vs Etiology and Risk Factors | 0.169 | 1.0000 | N |
| GFOG | Content category | Diagnosis and Screening vs Prevention and Patient Education | 4.410 | 1.033e-04 | Y |
| GFOG | Content category | Diagnosis and Screening vs Treatment and Prognosis | 1.191 | 1.0000 | N |
| GFOG | Content category | Etiology and Risk Factors vs Prevention and Patient Education | 4.241 | 2.223e-04 | Y |
| GFOG | Content category | Etiology and Risk Factors vs Treatment and Prognosis | 1.022 | 1.0000 | N |
| GFOG | Content category | Prevention and Patient Education vs Treatment and Prognosis | -3.219 | 0.0129 | Y |
| FKGL | Model | Deep Seek vs Doubao | 0.150 | 1.0000 | N |
| FKGL | Model | Deep Seek vs GPT-5 | -1.403 | 1.0000 | N |
| FKGL | Model | Deep Seek vs Tongyi Qianwen | 0.104 | 1.0000 | N |
| FKGL | Model | Deep Seek vs Wenxin Yiyan | 1.913 | 0.5575 | N |
| FKGL | Model | Doubao vs GPT-5 | -1.553 | 1.0000 | N |
| FKGL | Model | Doubao vs Tongyi Qianwen | -0.046 | 1.0000 | N |
| FKGL | Model | Doubao vs Wenxin Yiyan | 1.763 | 0.7788 | N |
| FKGL | Model | GPT-5 vs Tongyi Qianwen | 1.507 | 1.0000 | N |
| FKGL | Model | GPT-5 vs Wenxin Yiyan | 3.316 | 0.0091 | Y |
| FKGL | Model | Tongyi Qianwen vs Wenxin Yiyan | 1.809 | 0.7038 | N |
| FKGL | Content category | Clinical Manifestations and Classification vs Diagnosis and Screening | -4.673 | 2.961e-05 | Y |
| FKGL | Content category | Clinical Manifestations and Classification vs Etiology and Risk Factors | -2.859 | 0.0426 | Y |
| FKGL | Content category | Clinical Manifestations and Classification vs Prevention and Patient Education | -0.965 | 1.0000 | N |
| FKGL | Content category | Clinical Manifestations and Classification vs Treatment and Prognosis | -2.363 | 0.1815 | N |
| FKGL | Content category | Diagnosis and Screening vs Etiology and Risk Factors | 1.815 | 0.6954 | N |
| FKGL | Content category | Diagnosis and Screening vs Prevention and Patient Education | 3.709 | 0.0021 | Y |
| FKGL | Content category | Diagnosis and Screening vs Treatment and Prognosis | 2.311 | 0.2084 | N |
| FKGL | Content category | Etiology and Risk Factors vs Prevention and Patient Education | 1.894 | 0.5824 | N |
| FKGL | Content category | Etiology and Risk Factors vs Treatment and Prognosis | 0.496 | 1.0000 | N |
| FKGL | Content category | Prevention and Patient Education vs Treatment and Prognosis | -1.398 | 1.0000 | N |
| CL | Model | Deep Seek vs Doubao | -2.156 | 0.3112 | N |
| CL | Model | Deep Seek vs GPT-5 | -2.820 | 0.0480 | Y |
| CL | Model | Deep Seek vs Tongyi Qianwen | -3.534 | 0.0041 | Y |
| CL | Model | Deep Seek vs Wenxin Yiyan | 0.867 | 1.0000 | N |
| CL | Model | Doubao vs GPT-5 | -0.665 | 1.0000 | N |
| CL | Model | Doubao vs Tongyi Qianwen | -1.379 | 1.0000 | N |
| CL | Model | Doubao vs Wenxin Yiyan | 3.022 | 0.0251 | Y |
| CL | Model | GPT-5 vs Tongyi Qianwen | -0.714 | 1.0000 | N |
| CL | Model | GPT-5 vs Wenxin Yiyan | 3.687 | 0.0023 | Y |
| CL | Model | Tongyi Qianwen vs Wenxin Yiyan | 4.401 | 1.078e-04 | Y |
| CL | Content category | Clinical Manifestations and Classification vs Diagnosis and Screening | -3.567 | 0.0036 | Y |
| CL | Content category | Clinical Manifestations and Classification vs Etiology and Risk Factors | -3.052 | 0.0227 | Y |
| CL | Content category | Clinical Manifestations and Classification vs Prevention and Patient Education | -0.049 | 1.0000 | N |
| CL | Content category | Clinical Manifestations and Classification vs Treatment and Prognosis | -1.875 | 0.6082 | N |
| CL | Content category | Diagnosis and Screening vs Etiology and Risk Factors | 0.515 | 1.0000 | N |
| CL | Content category | Diagnosis and Screening vs Prevention and Patient Education | 3.518 | 0.0043 | Y |
| CL | Content category | Diagnosis and Screening vs Treatment and Prognosis | 1.692 | 0.9060 | N |
| CL | Content category | Etiology and Risk Factors vs Prevention and Patient Education | 3.003 | 0.0267 | Y |
| CL | Content category | Etiology and Risk Factors vs Treatment and Prognosis | 1.177 | 1.0000 | N |
| CL | Content category | Prevention and Patient Education vs Treatment and Prognosis | -1.826 | 0.6788 | N |
| SMOG | Model | Deep Seek vs Doubao | -0.033 | 1.0000 | N |
| SMOG | Model | Deep Seek vs GPT-5 | -1.608 | 1.0000 | N |
| SMOG | Model | Deep Seek vs Tongyi Qianwen | 0.529 | 1.0000 | N |
| SMOG | Model | Deep Seek vs Wenxin Yiyan | 1.944 | 0.5192 | N |
| SMOG | Model | Doubao vs GPT-5 | -1.576 | 1.0000 | N |
| SMOG | Model | Doubao vs Tongyi Qianwen | 0.562 | 1.0000 | N |
| SMOG | Model | Doubao vs Wenxin Yiyan | 1.977 | 0.4810 | N |
| SMOG | Model | GPT-5 vs Tongyi Qianwen | 2.137 | 0.3257 | N |
| SMOG | Model | GPT-5 vs Wenxin Yiyan | 3.552 | 0.0038 | Y |
| SMOG | Model | Tongyi Qianwen vs Wenxin Yiyan | 1.415 | 1.0000 | N |
| SMOG | Content category | Clinical Manifestations and Classification vs Diagnosis and Screening | -5.044 | 4.570e-06 | Y |
| SMOG | Content category | Clinical Manifestations and Classification vs Etiology and Risk Factors | -4.021 | 5.790e-04 | Y |
| SMOG | Content category | Clinical Manifestations and Classification vs Prevention and Patient Education | -1.810 | 0.7026 | N |
| SMOG | Content category | Clinical Manifestations and Classification vs Treatment and Prognosis | -3.588 | 0.0033 | Y |
| SMOG | Content category | Diagnosis and Screening vs Etiology and Risk Factors | 1.022 | 1.0000 | N |
| SMOG | Content category | Diagnosis and Screening vs Prevention and Patient Education | 3.233 | 0.0122 | Y |
| SMOG | Content category | Diagnosis and Screening vs Treatment and Prognosis | 1.456 | 1.0000 | N |
| SMOG | Content category | Etiology and Risk Factors vs Prevention and Patient Education | 2.211 | 0.2704 | N |
| SMOG | Content category | Etiology and Risk Factors vs Treatment and Prognosis | 0.433 | 1.0000 | N |
| SMOG | Content category | Prevention and Patient Education vs Treatment and Prognosis | -1.778 | 0.7548 | N |
| LW | Model | Deep Seek vs Doubao | 2.031 | 0.4224 | N |
| LW | Model | Deep Seek vs GPT-5 | 1.564 | 1.0000 | N |
| LW | Model | Deep Seek vs Tongyi Qianwen | 0.762 | 1.0000 | N |
| LW | Model | Deep Seek vs Wenxin Yiyan | -0.262 | 1.0000 | N |
| LW | Model | Doubao vs GPT-5 | -0.467 | 1.0000 | N |
| LW | Model | Doubao vs Tongyi Qianwen | -1.269 | 1.0000 | N |
| LW | Model | Doubao vs Wenxin Yiyan | -2.293 | 0.2183 | N |
| LW | Model | GPT-5 vs Tongyi Qianwen | -0.803 | 1.0000 | N |
| LW | Model | GPT-5 vs Wenxin Yiyan | -1.826 | 0.6779 | N |
| LW | Model | Tongyi Qianwen vs Wenxin Yiyan | -1.024 | 1.0000 | N |
| LW | Content category | Clinical Manifestations and Classification vs Diagnosis and Screening | 3.484 | 0.0049 | Y |
| LW | Content category | Clinical Manifestations and Classification vs Etiology and Risk Factors | 1.245 | 1.0000 | N |
| LW | Content category | Clinical Manifestations and Classification vs Prevention and Patient Education | 0.915 | 1.0000 | N |
| LW | Content category | Clinical Manifestations and Classification vs Treatment and Prognosis | 4.376 | 1.207e-04 | Y |
| LW | Content category | Diagnosis and Screening vs Etiology and Risk Factors | -2.239 | 0.2518 | N |
| LW | Content category | Diagnosis and Screening vs Prevention and Patient Education | -2.569 | 0.1020 | N |
| LW | Content category | Diagnosis and Screening vs Treatment and Prognosis | 0.893 | 1.0000 | N |
| LW | Content category | Etiology and Risk Factors vs Prevention and Patient Education | -0.330 | 1.0000 | N |
| LW | Content category | Etiology and Risk Factors vs Treatment and Prognosis | 3.131 | 0.0174 | Y |
| LW | Content category | Prevention and Patient Education vs Treatment and Prognosis | 3.462 | 0.0054 | Y |

**Supplementary Table S3. Correlation coefficients (Pearson’s r) and P values across all outputs (n = 100)**

Bonferroni adjustment is applied across all variable pairs reported in this table (m = 36).

| **Variable 1** | **Variable 2** | **Pearson’s r** | **P value (two-tailed)** | **Bonferroni-adjusted P** | **Significant (Y/N)** |
| --- | --- | --- | --- | --- | --- |
| GQS | c-PEMAT-P | 0.149 | 0.1401 | 1.0000 | N |
| GQS | ARI | 0.230 | 0.0215 | 0.7756 | N |
| GQS | FRES | -0.149 | 0.1403 | 1.0000 | N |
| GQS | GFOG | 0.124 | 0.2205 | 1.0000 | N |
| GQS | FKGL | 0.198 | 0.0481 | 1.0000 | N |
| GQS | CLI | 0.116 | 0.2489 | 1.0000 | N |
| GQS | SMOG | 0.285 | 0.0041 | 0.1480 | N |
| GQS | LW | -0.105 | 0.2999 | 1.0000 | N |
| c-PEMAT-P | ARI | 0.155 | 0.1233 | 1.0000 | N |
| c-PEMAT-P | FRES | -0.014 | 0.8867 | 1.0000 | N |
| c-PEMAT-P | GFOG | 0.015 | 0.8823 | 1.0000 | N |
| c-PEMAT-P | FKGL | 0.066 | 0.5163 | 1.0000 | N |
| c-PEMAT-P | CLI | 0.090 | 0.3744 | 1.0000 | N |
| c-PEMAT-P | SMOG | 0.070 | 0.4880 | 1.0000 | N |
| c-PEMAT-P | LW | 0.017 | 0.8632 | 1.0000 | N |
| ARI | FRES | -0.757 | 8.427e-20 | 3.034e-18 | Y |
| ARI | GFOG | 0.614 | 1.134e-11 | 4.081e-10 | Y |
| ARI | FKGL | 0.923 | 2.576e-42 | 9.273e-41 | Y |
| ARI | CLI | 0.644 | 5.114e-13 | 1.841e-11 | Y |
| ARI | SMOG | 0.828 | 2.196e-26 | 7.905e-25 | Y |
| ARI | LW | -0.637 | 1.042e-12 | 3.752e-11 | Y |
| FRES | GFOG | -0.852 | 2.718e-29 | 9.785e-28 | Y |
| FRES | FKGL | -0.891 | 2.502e-35 | 9.007e-34 | Y |
| FRES | CLI | -0.894 | 5.035e-36 | 1.813e-34 | Y |
| FRES | SMOG | -0.785 | 4.610e-22 | 1.660e-20 | Y |
| FRES | LW | 0.635 | 1.282e-12 | 4.615e-11 | Y |
| GFOG | FKGL | 0.722 | 2.415e-17 | 8.694e-16 | Y |
| GFOG | CLI | 0.794 | 5.986e-23 | 2.155e-21 | Y |
| GFOG | SMOG | 0.783 | 6.523e-22 | 2.348e-20 | Y |
| GFOG | LW | -0.505 | 8.677e-08 | 3.124e-06 | Y |
| FKGL | CLI | 0.676 | 1.148e-14 | 4.132e-13 | Y |
| FKGL | SMOG | 0.882 | 9.113e-34 | 3.281e-32 | Y |
| FKGL | LW | -0.654 | 1.646e-13 | 5.926e-12 | Y |
| CLI | SMOG | 0.617 | 7.818e-12 | 2.815e-10 | Y |
| CLI | LW | -0.569 | 6.378e-10 | 2.296e-08 | Y |
| SMOG | LW | -0.637 | 1.090e-12 | 3.924e-11 | Y |

**Supplementary Table S4. Proportion of outputs meeting readability cutoffs (exploratory), by model**

Cutoffs are reported for comparability with prior grade-level literature; interpret as exploratory for Chinese-language outputs.

| **Model** | **n outputs** | **% FKGL ≤ 8** | **% FKGL ≤ 6** | **% SMOG ≤ 8 (optional)** |
| --- | --- | --- | --- | --- |
| DeepSeek | 20.0 | 0.0 | 0.0 | 0.0 |
| Doubao | 20.0 | 0.0 | 0.0 | 0.0 |
| GPT-5 | 20.0 | 0.0 | 0.0 | 0.0 |
| Tongyi Qianwen | 20.0 | 0.0 | 0.0 | 0.0 |
| Wenxin Yiyan | 20.0 | 0.0 | 0.0 | 0.0 |
| Overall | 100.0 | 0.0 | 0.0 | 0.0 |

**Supplementary Table S5. Proportion of outputs meeting readability cutoffs (exploratory), by content category**

Cutoffs are reported for comparability with prior grade-level literature; interpret as exploratory for Chinese-language outputs.

| **Category** | **n outputs** | **% FKGL ≤ 8** | **% FKGL ≤ 6** | **% SMOG ≤ 8 (optional)** |
| --- | --- | --- | --- | --- |
| Clinical Manifestations and Classification | 20.0 | 0.0 | 0.0 | 0.0 |
| Diagnosis and Screening | 20.0 | 0.0 | 0.0 | 0.0 |
| Etiology and Risk Factors | 20.0 | 0.0 | 0.0 | 0.0 |
| Prevention and Patient Education | 20.0 | 0.0 | 0.0 | 0.0 |
| Treatment and Prognosis | 20.0 | 0.0 | 0.0 | 0.0 |
| Overall | 100.0 | 0.0 | 0.0 | 0.0 |
