# Supplementary figures and images for: Decoupled quality and readability in skin cancer education from large language models
Source: Front Public Health. 2026 Feb 20;14:1777577. doi: 10.3389/fpubh.2026.1777577 (PMC12962940; doi:10.3389/fpubh.2026.1777577)

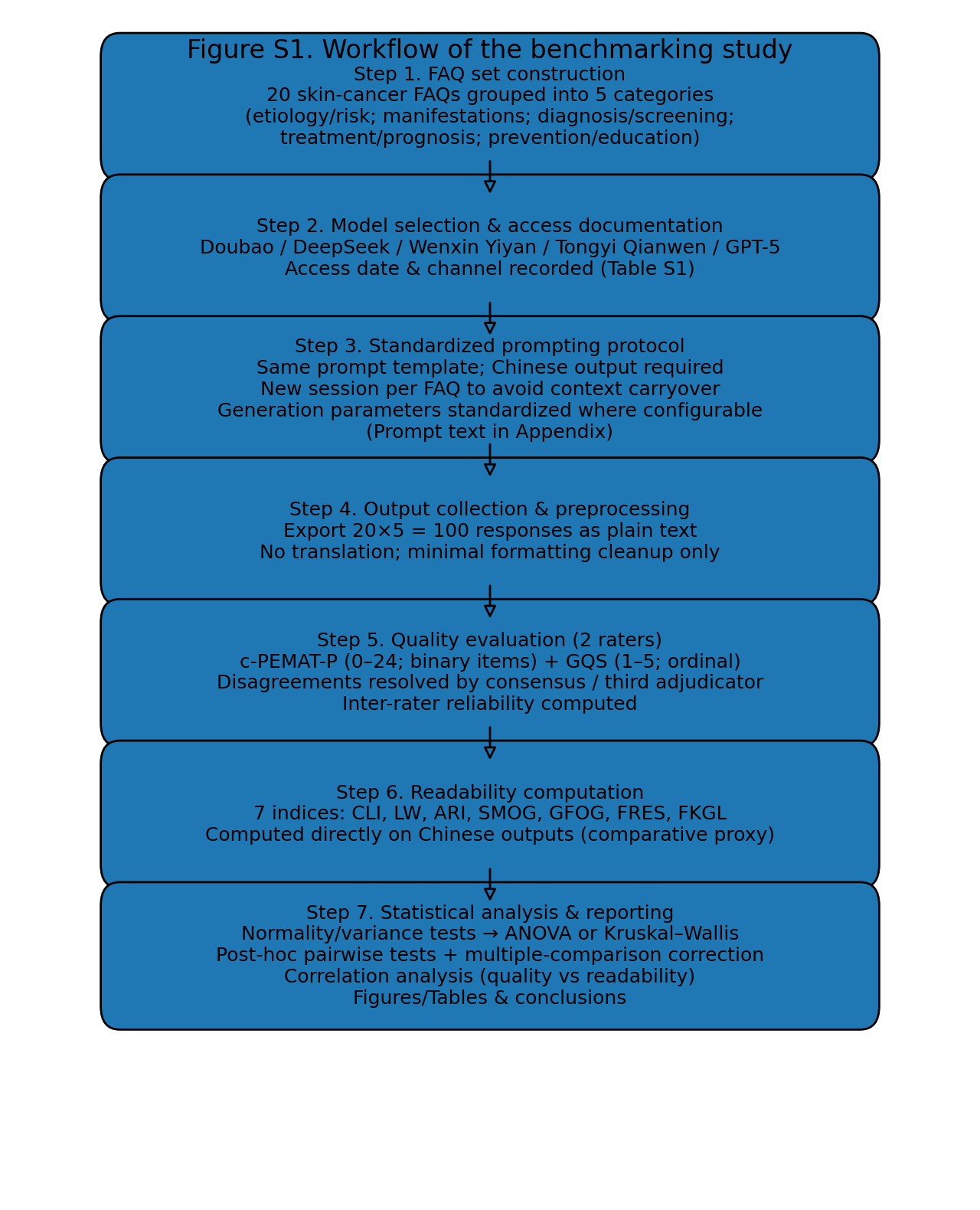

Supplement: Supplementary file 3 [file Image_1.png]
